# A Comparison of Pattern of Pregnancy Loss in Women with Infertility Undergoing IVF and Women with Unexplained Recurrent Miscarriages Who Conceive Spontaneously

**DOI:** 10.1155/2015/989454

**Published:** 2015-10-20

**Authors:** Vidya A. Tamhankar, Beiyu Liu, Junhao Yan, Tin-Chiu Li

**Affiliations:** ^1^Department of Reproductive Medicine, University of Sheffield, Sheffield Teaching Hospitals, Jessop Wing, Tree Root Walk, Sheffield S10 2SF, UK; ^2^Department of Obstetrics and Gynecology, Bronx-Lebanon Hospital Center, Albert Einstein College of Medicine, The Bronx, NY 10457, USA; ^3^Department of Obstetrics and Gynaecology, Chinese University of Hong Kong, Prince of Wales Hospital, Shatin, N.T., Hong Kong

## Abstract

*Objective*. Women with infertility and recurrent miscarriages may have an overlapping etiology. The aim of this study was to compare the pregnancy loss in pregnancies after IVF treatment with spontaneous pregnancies in women with recurrent miscarriages and to assess differences related to cause of infertility. *Methods*. The outcome from 1220 IVF pregnancies (Group I) was compared with 611 spontaneous pregnancies (Group II) in women with recurrent miscarriages. Subgroup analysis was performed in Group I based on cause of infertility: tubal factor (392 pregnancies); male factor (610 pregnancies); and unexplained infertility (218 pregnancies). *Results*. The clinical pregnancy loss rate in Group I (14.3%) was significantly lower than that of Group II (25.8%, *p* < 0.001) and this was independent of the cause of infertility. However the timing of pregnancy loss was similar between Groups I and II. The clinical pregnancy loss rate in Group I was similar in different causes of infertility. *Conclusions*. The clinical pregnancy loss rate following IVF treatment is lower than that of women with unexplained recurrent miscarriages who conceived spontaneously. This difference persists whether the infertility is secondary to tubal factors, male factors, or unexplained cause.

## 1. Introduction

Infertility and miscarriage are two facets of reproductive failure that are said to have overlapping etiologies. A number of pathologies have been considered to be associated with both infertility and miscarriage, namely, polycystic ovarian disease [1], uterine septum, and uterine fibroid [2]. A higher prevalence of infertility has also been detected among patients with recurrent miscarriage [3].

The risk of miscarriage in women with infertility has been reported to range widely from 7% [4] to as high as 70% [5]. There are several explanations for these differences reported by various investigators. Firstly, the definition of miscarriage used by various investigators is quite different; some included biochemical losses while others reported only clinical pregnancy loss. Secondly, infertility itself is a rather heterogeneous condition with different underlying causes. It is possible that the miscarriage rate in women with different underlying causes of infertility may be different. Very few investigators have, however, examined the impact of infertility diagnosis on the miscarriage rate. Miscarriage rates are said to be the highest among PCOS as compared to other groups of infertility [6]. Thirdly, it is possible that infertility treatment itself may influence the likelihood of miscarriage. It has been reported that pregnancy following IVF treatment has a particularly high loss rate, with several reports on rates well over 30% [7], similar to that observed in women with a history of recurrent miscarriage.

Miscarriage may occur at different stages of the pregnancy, for example, biochemical loss prior to any ultrasound evidence of an intrauterine gestational sac, early clinical loss in which there is an ultrasound evidence of an intrauterine sac, fetal loss in first trimester after demonstration of fetal pole and heart beats, and second trimester loss. It is possible that various underlying pathology results in loss at different stages of the pregnancy. Patients with recurrent miscarriage and infertility are at a high risk of pregnancy loss. A similar pattern of miscarriage among these two cohorts of patients may indicate common pathogenesis of pregnancy loss in them. To the best of our knowledge, there has been no study that has compared the miscarriage rate between patients with recurrent miscarriage and infertility. Miscarriage rate among naturally conceived pregnancies is particularly difficult to measure and often underreported. As patients with recurrent miscarriage are closely monitored in our unit, this hurdle is easily overcome. The objective of our study is to compare the rate and pattern of miscarriage between spontaneous conceptions in women with history of recurrent miscarriage and IVF pregnancies in women with infertility.

## 2. Methods

The study was conducted at the Reproductive Medicine Unit, Jessop Wing, Sheffield. Women were included in the study from the recurrent miscarriage clinic and assisted conception unit. Data was retrospectively analyzed from a prospectively maintained database in the unit. Ethics approval was not required as patient identifying details were excluded from database.

### 2.1. Subjects

Two groups of subjects were included in the study.

Group I consisted of infertile women, who conceived following IVF ± ICSI treatment, for either tubal factor (Group IA), male factor (Group IB), or unexplained infertility (Group IC). Tubal factor (Group IA) was defined as patients who were unable to conceive due to tubal disease diagnosed by hysterosalpingogram (HSG) or laparoscopy. Male factor infertility (Group IB) was defined when there was abnormal semen analysis as per WHO 2010 criteria [8]. Unexplained infertility (Group IC) was defined when the basic infertility evaluation including midluteal progesterone, hysterosalpingogram, ultrasonography, and semen analysis all showed normal results. Out of 962 women in Group I who conceived with IVF, 144 women had at least one miscarriage previously and a quarter of these women had 2 miscarriages. None of these women had three or more miscarriages. The total number of previous miscarriages was 189 in this group giving a mean miscarriage rate (+SD) of 0.2 ± 0.4 per person.

Group II included women who conceived spontaneously with history of recurrent miscarriage of unknown etiology. Unexplained recurrent miscarriage was defined as subjects who had 3 or more consecutive miscarriages with no evidence of endocrine, immunological, anatomical, and genetic cause for their recurrent pregnancy loss following investigations according to an established protocol [9]. The investigations they had included karyotyping for both partners, antiphospholipid antibody (lupus anticoagulant, anticardiolipin antibody, and beta-2 glycoprotein) and thrombophilia screen, thyroid function test, ultrasonography, and hysterosalpingogram. Unexplained recurrent miscarriage was a diagnosis of exclusion. The mean number of miscarriages in this group was 3.4 ± 0.6.

### 2.2. Exclusion Criteria

All patients above the age of 37 years were excluded from the study in order to reduce the confounding variable of age. IVF pregnancies involving donor gametes were also excluded from the study. Patients having IVF for etiologies other than tubal, male, or unexplained infertility were excluded. Biochemical pregnancy loss, multiple pregnancies, ectopic pregnancies, and pregnancies terminated for social reasons were excluded from analysis.

## 3. Treatment

Group I had in vitro fertilization (IVF) or intracytoplasmic sperm injection (ICSI) treatment depending on the clinical indication. As a default protocol, controlled ovarian stimulation with human recombinant FSH and GnRH antagonist was commenced in the follicular phase of the menstrual cycle. In some women, with poor ovarian reserve, GnRH agonist was used as a flare-up protocol. Serial ultrasound examinations and serum estradiol measurement were used to assess the ovarian response. When a minimum of 3 follicles reached a size of ≥17 mm, ovulation was triggered with human chorionic gonadotropin and oocyte retrieval was performed 36 hours later using standard ultrasound guided transvaginal approach. Collected oocytes were fertilized in vitro using IVF or ICSI as clinically appropriate. Progesterone was used for luteal phase support for 2 weeks in the form of vaginal pessaries and was discontinued on the day of pregnancy test as per the unit protocol. Embryo replacement was performed between days two and five. Pregnancy was diagnosed if plasma *β*HCG > 20 IU/L fourteen days after oocyte retrieval. Women with a positive pregnancy test were then followed up with serial Beta HCG assays and transvaginal scan at 6 weeks.

Group II women were seen in the recurrent miscarriage clinic within a week of positive home urine pregnancy test. They all received pregnancy support through the early pregnancy clinic. Follow-up was similar to Group I with serial Beta HCG assays and transvaginal scan at 6 weeks. None of these patients received any empirical treatment in the form of progesterone support or aspirin.

### 3.1. Pregnancy Outcome

Outcome was categorized into 6 groups as follows:

- Biochemical loss was defined when the Beta HCG values were higher than 20 IU/L with no ultrasound evidence of pregnancy.
- Ectopic pregnancy was defined when ultrasound or laparoscopy confirmed the presence of ectopic gestation or when Beta HCG values were more than 1000 IU/L with no ultrasound evidence of intrauterine gestation.
- Clinical pregnancy was defined when there was positive pregnancy test accompanied by ultrasound evidence of intrauterine pregnancy.
- Embryonic loss was defined as pregnancy loss after the presence of intrauterine gestational sac but prior to demonstration of fetal heart beats.
- Fetal loss was defined as pregnancy loss after fetal heart beats had been detected, but before 13 weeks of gestation.
- Second trimester loss was defined as pregnancy loss beyond 13 weeks of gestation.

Conceptions without ultrasound evidence of intrauterine pregnancy (biochemical loss and ectopic) were excluded from the analysis.

Data in the two groups were compared with appropriate statistical test (Fisher's exact test, Student's *t*-test, and ANOVA one-way test) using GraphPad InStat version 3.10, GraphPad Software, San Diego, CA, USA, and SPSS for Windows, Rel. 20.0.0. 2011., SPSS Inc., Chicago.

## 4. Results

A total of 962 women who conceived after IVF treatment (Group I) and 368 women with unexplained recurrent miscarriage (Group II) were included in the study. Their demographic data is presented in Table 1. All groups were comparable with regard to mean age and BMI.

**Figure 1:**
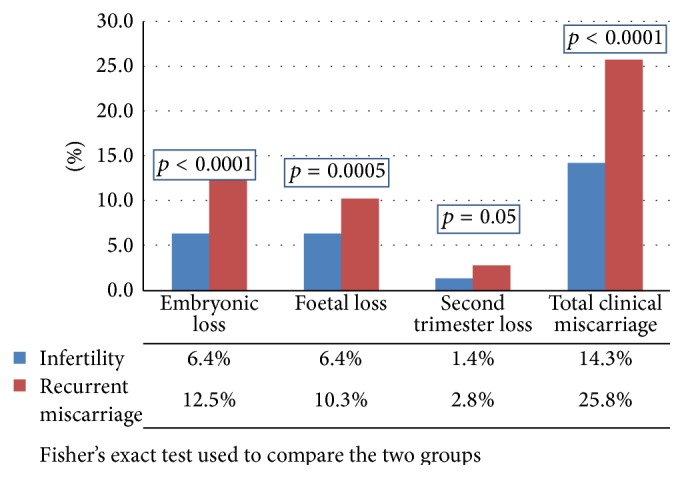
A comparison of the rates of pregnancy loss between women with infertility and recurrent miscarriage.

There were 1220 pregnancies in Group I. This was further divided into Groups IA, IB, and IC depending on the etiology (tubal, male, and unexplained). All women in these groups conceived with either IVF treatment (*n* = 644) or IVF + ICSI (*n* = 576). There were 611 pregnancies in Group II. These women had history of three of more previous miscarriages of unexplained etiology. In Figure 1, the rates of pregnancy loss between Group I and Group II are compared. The total clinical pregnancy loss in women with recurrent miscarriage (25.8%) was significantly higher than that of women with infertility who conceived following IVF treatment (14.3%). The embryonic loss rate, fetal loss rate, and second trimester loss rate were all significantly higher in women with recurrent miscarriage.

The pregnancy loss in subgroups IA, IB, and IC was individually compared to Group II yielding similar results, namely, Group IA (14.4% versus 25.8%; *p* < 0.001); Group IB (13.5% versus 25.8%; *p* < 0.001); and Group IC (15.9% versus 25.8%; *p* < 0.002).

**Figure 2:**
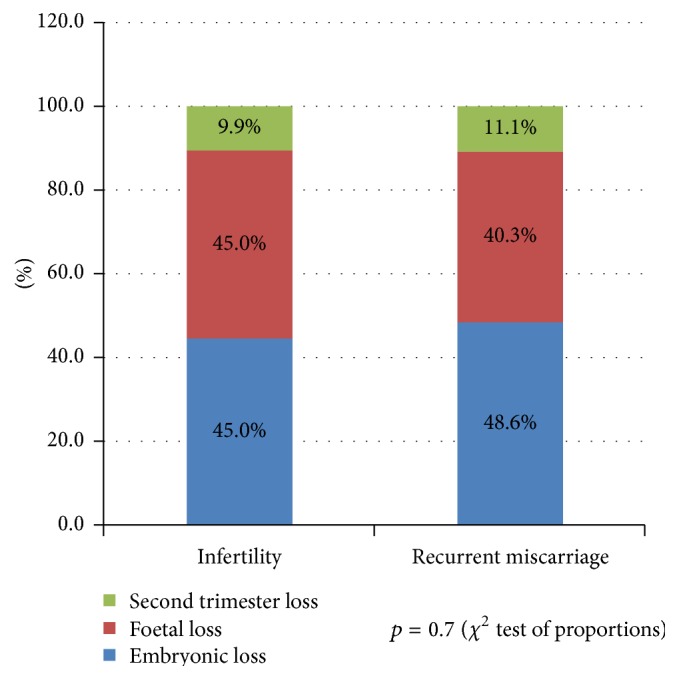
A comparison of the pattern of pregnancy loss between women with infertility and recurrent miscarriage.

In Figure 2, the pattern of pregnancy loss in both groups was compared. There was no difference between the two groups (*p* = 0.7).

**Figure 3:**
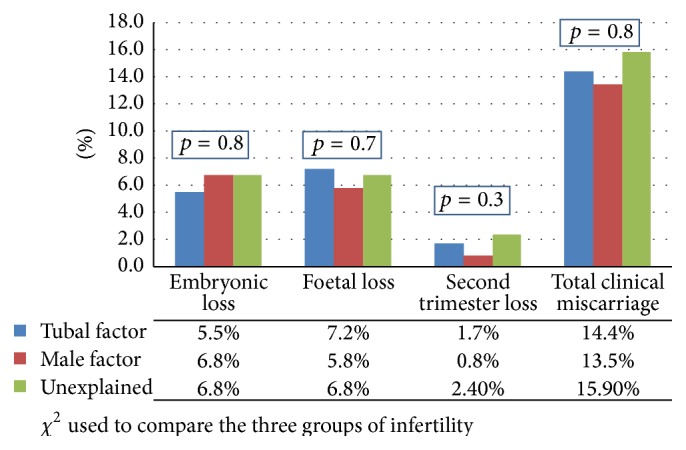
A comparison of the rates of pregnancy loss in tubal, male, and unexplained infertility.

**Figure 4:**
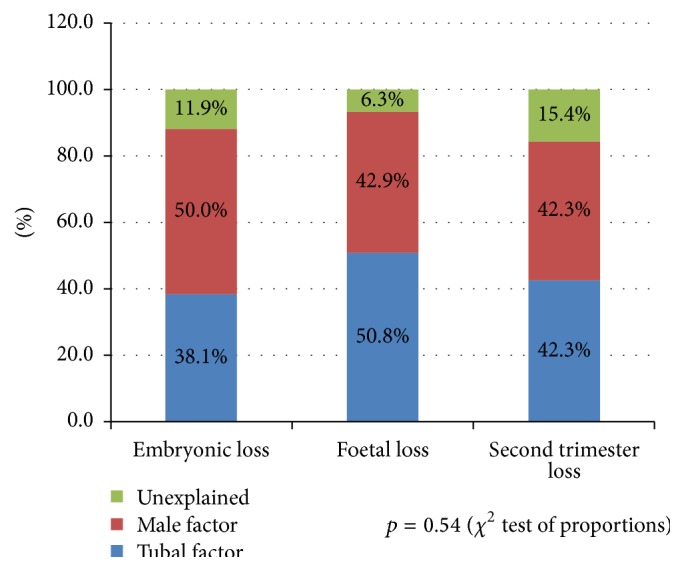
A comparison of the pattern of miscarriage in tubal, male, and unexplained infertility.

In Figure 3, the pregnancy loss among the three subgroups of women with infertility (tubal, male, and unexplained) is compared. There was no difference in the rate of pregnancy loss among the three groups. In Figure 4, the pattern of pregnancy loss among the three groups was compared. Whilst embryonic loss in male infertility appeared highest among the three groups and fetal loss appeared highest in tubal infertility, the difference did not reach statistical significance (*p* = 0.7).

## 5. Discussion

In this study we compared the rate and pattern of miscarriage in two groups of women with reproductive failure, namely, women with recurrent miscarriage who conceived spontaneously and women with infertility who conceived following IVF treatment. We found significant differences between the pregnancy losses in these two groups. Studies have assessed pregnancy loss after IVF and in recurrent miscarriage patients, but no direct comparison has been made [10–13]. We performed this comparative study between these groups as common etiological factors exist for pregnancy loss in both of these groups [14].

### 5.1. Biochemical Loss

In our study, the biochemical loss rate was 22.5% among Group I who had IVF/ICSI treatment and 5% in Group II who conceived spontaneously. This is consistent with previous studies, where the biochemical loss rate has been reported to range from 22% to 31% [15–17] after IVF, which appears to be higher than the rate reported in women who conceive naturally which ranges from 8% to 22% [12, 18]. The apparently higher rate of biochemical loss in the former group could be a consequence of increased surveillance of women undergoing IVF and hence this has been excluded from the study. In most IVF units, Beta HCG is measured 14 days after oocyte retrieval with the result that biochemical pregnancy is more likely to be detected. This is in contrast to women with recurrent miscarriage who wait until a few days after a missed period to do urine pregnancy test. By this time a significant proportion of biochemical pregnancy may have escaped detection. Hence to make a valid comparison of the biochemical loss rate between spontaneous conceptions in recurrent miscarriage and IVF pregnancies in infertility, the same method of surveillance is required for both groups, namely, measurement of serum BHCG 14 days after ovulation or oocyte retrieval. In this study, therefore, we have limited our comparison to clinical pregnancy loss which is after ultrasound confirmation of intrauterine pregnancy.

Biochemical pregnancies represent conceptions that have started to implant but fail to progress at a very early stage before clinical diagnosis of pregnancy is established. Endometrial factors, maternal age, stress, and sperm DNA fragmentation have been proposed as possible aetiological mechanisms for biochemical pregnancy [19]. Several studies have shown that biochemical pregnancies have positive prognosis on the outcome of subsequent IVF cycles [20–22]. Although we have not included biochemical pregnancies in our analysis due to methodological issues, they have prognostic significance for subsequent pregnancy outcome.

### 5.2. Clinical Miscarriages

We found that the overall clinical loss in the unexplained recurrent miscarriage group (25.8%) was significantly higher (*p* = 0.001) than the infertility group (14.3%). The rates observed in these two groups of subjects both appear to be higher than the rate observed in the general population (7.9% to 13.5%) [23, 24].

*First Trimester Loss*. Embryonic loss was significantly higher in recurrent miscarriage than that of the infertility group (12.5% versus 6.4%; *p* < 0.0001). The rate of fetal loss per clinical pregnancy in women with recurrent miscarriage (10.3%) was also significantly higher (*p* = 0.03) than that (6.4%) of the infertile group. A review of the literature has shown that pregnancy loss rate after documentation of fetal heart (fetal loss) was 3–6% in patients with no history of infertility and/or recurrent miscarriages [25–27]. In the infertile populations this ranges from 7 to 15% [28–30]. However, in women with a history of recurrent miscarriage, pregnancy loss after ultrasound documentation of fetal heart beats (fetal loss rate) has been shown to be higher, at 17–22% [31, 32]. Our observation in this study on fetal loss is consistent with earlier reports.

*Second Trimester Loss*. After the first trimester the pregnancy loss in the recurrent miscarriage group was 2.8% which represents a twofold increase in comparison with the infertility group (1.4%). This difference did not quite reach statistical significance (*p* = 0.05). This is almost certainly due to type 2 error. Verifying a significant difference between the two groups requires studies with larger sample sizes.

### 5.3. Spontaneous Conceptions versus IVF Treatment

One possible criticism of the study is that we compared the two groups of women who conceived with different methods, namely, spontaneous conception for women with recurrent miscarriage and IVF conception for women with infertility. Ideally comparison should be made when the method of conception is the same for two different groups of women. However women with recurrent pregnancy loss do not usually have a problem with conception and very few require assisted conception. Alternately one may compare only natural pregnancies in both groups, but women with infertility often require treatment to achieve conception. In our study the comparison between the two groups has been made on the assumption that IVF does not in itself alter the likelihood of clinical loss when compared with natural conception. Though limited information is available, the study by Schieve et al. has demonstrated that IVF does not increase the risk of clinical pregnancy loss when compared to natural conception [33].

### 5.4. Age as a Confounding Variable

It is well recognized that older women are more likely to have pregnancy loss. Hence to minimize the impact of age as a confounding variable we have excluded women over the age of 37 years in our study. Our data showed no significant difference in mean ages between women with recurrent miscarriage and different groups of infertility.

*Comparison among Different Groups of Infertility*. Our study has shown no significant differences in the rate of pregnancy loss among the three groups of infertility (Figure 3). The embryonic loss appeared highest in the male infertility and lowest in the tubal infertility. The pregnancy loss in the second trimester was highest in unexplained infertility and lowest in male infertility. These differences though did not reach statistical significance (*p* = 0.54). This is in contrast to the study by Omland et al. where the first trimester miscarriage rate and particularly those before 6 weeks of pregnancy were considerably lower in unexplained infertility in comparison with endometriosis and tubal factor [34].

To conclude, we found that women with unexplained recurrent miscarriage who conceived spontaneously have a higher risk of pregnancy loss than women who conceived after IVF treatment for tubal factor, male factor, or unexplained infertility. However the pattern of pregnancy loss remained the same. The rate and pattern of pregnancy loss following IVF were similar irrespective of the etiology of infertility.

## Figures and Tables

**Table 1:** Demographic data of women with recurrent miscarriage and infertility.

	Infertility				(II) Unexplained recurrent miscarriage	*p* value
	(I) All infertility factors	(IA) Tubal infertility	(IB) Male infertility	(IC) Unexplained infertility		
Patients	962	304	473	185	368	
Conception cycles (*n*)	1220	392	610	218	611	
Body Mass Index, kg/m^2^ (mean ± SD)	24.4 ± 3.8	24.7 ± 3.7	24.7 ± 4.1	23.8 ± 3.6	25.4 ± 4.9	*p* = 0.092^*∗*^
Age, years (mean ± SD)	32.5 ± 4.1	32.68 ± 4.1	32.2 ± 4.5	32.9 ± 3.1	32.1 ± 4.4	*p* = 0.15^*∗*^

^*∗*^ANOVA used to compare means between different groups.
